# Thanksgiving

**Published:** 1914-11

**Authors:** Ruth Sterry


### EDITORIAL DEPARTMENT.

MRS. F. E. DANIEL, Publisher and Managing Editor.
DR. R. H. L. BIBB, Associate Editor.

CONTRIBUTING EDITORS:

Dr. H. Sheridan Baketel, New York, editor *Medical Times:* Dr. M. H. Boerner, Austin, Texas; Dr. G. Henri Bogart, Paris, Ill.; Dr. M. M. Carrick, Dallas, Texas; Dr. Edward H. Carey, Dean Medical Department, Baylor University, Dallas, Texas; Dr. W. L. Crosthwaite, Waco, Texas; Dr. T. D. Crothers, Hartford, Conn.; Dr. H. W. Cummings, Hearne, Texas; Dr. Oscar Dowling, New Orleans, President Louisiana State Board of Health; Havelock Ellis, M. D., London, Eng.; Dr. May Agnes Hopkins, Dallas, Texas; Dr. A. W. Fly, Galveston, Texas; Dr. G. B. Foscue, Waco, Texas; Dr. E. S. Goodhue, Holualoa, Kona, Hawaii; Dr. M. B. Grace, Seguin, Texas; Dr. Joe Gilbert, Austin, Texas; Dr. Charles W. Hughes, St. Louis, editor *Alienist and Neurologist*; Dr. James G. Kiernan, Chicago; Dr. George H. Lee, Galveston, Texas; Dr. John T. Moore, Houston, Texas; Dr. Frank Paschai, San Antonio, Texas; Dr. John Preston, Austin, Texas, Superintendent State Lunatic Asylum; Dr. Bacon Saunders, Fort Worth, Texas; Dr. Allen J. Smith, Philadelphia, Medical Department, University of Pennsylvania; Dr. J. T. Scott, Austin, Texas; Dr. Ralph Steiner, Austin, Texas, President Texas State Board of Health; Dr. John S. Turner, Dallas, Texas; Dr. Charles Venable, San Antonio, Texas.

### Thanksgiving.

BY RUTH STERRY.

Not what we have, O Lord, but what we missed:
For shining eyes tonight Death might have kissed,
For loving hands so dear we might not hold,
For lips we love which might tonight be cold.

For what we missed, O Lord, for what we missed:
The child who might have wandered, Judas kissed,
The sin which might have found us unaware
And entering in our hearts have flourished there.

For what we missed, O Lord, for what we missed
We give Thee thanks; for days no blight has kissed—
For hearts and homes tonight that by Thy grace
Rejoice that there is not an empty place.

